# Thyroid nodule malignancy is associated with increased non-invasive hepatic fibrosis scores in metabolic subjects

**DOI:** 10.3389/fonc.2023.1233083

**Published:** 2023-10-26

**Authors:** Lucilla Crudele, Fabio Novielli, Carlo De Matteis, Stefano Petruzzelli, Patrizia Suppressa, Elsa Berardi, Gianfranco Antonica, Giuseppina Piazzolla, Carlo Sabbà, Giusi Graziano, Antonio Moschetta

**Affiliations:** ^1^ Department of Interdisciplinary Medicine, University of Bari “Aldo Moro”, Bari, Italy; ^2^ Center for Outcomes Research and Clinical Epidemiology (CORESEARCH), Pescara, Italy; ^3^ IINBB National Institute for Biostructure and Biosystems, Roma, Italy

**Keywords:** malignant thyroid nodule, liver fibrosis, thyroid cancer, BMI, obesity

## Abstract

**Introduction:**

Thyroid cancer incidence is increasing, and adiposity-related conditions are gaining space in its pathogenesis. In this study, we aimed to detect any anthropometric, biohumoral, and clinical features that might be associated with thyroid nodule malignancy, potentially representing novel non-invasive markers of thyroid cancer.

**Materials and methods:**

The study was conducted in a group of 142 consecutive outpatients (47 men and 95 women) who underwent fine-needle aspiration biopsy/cytology (FNAB/C) due to suspicion of malignancy from January 2018 to September 2022. We compared lipid and glycemic blood profiles as well as non-invasive liver fibrosis indexes such as aspartate aminotransferase (AST) to alanine aminotransferase (ALT) ratio (AAR), AST to platelet ratio index (APRI), and fibrosis index based on four factors (FIB-4) between patients with benign and malignant newly diagnosed nodules. Then, we performed receiver operating characteristic (ROC) analysis to assess their best cutoff values for discrimination of malignant nodules and chi-squared test to evaluate the association of specific dysmetabolic conditions with malignancy. To understand whether and to what degree dysmetabolic conditions increased the risk of thyroid nodule malignancy, we also calculated the odds ratio (OR) of the main biomarkers.

**Results:**

After FNAB/C, 121 (85%) patients were diagnosed with benign thyroid nodules, while 21 (15%) individuals were diagnosed with thyroid cancer. Comparing patients with benign and malignant nodules, we found that individuals with thyroid cancer exhibited increased body mass index (BMI) (p = 0.048) and fasting plasma glucose (p = 0.046). Intriguingly, considering non-invasive scores for liver fibrosis, subjects with thyroid cancer presented increased AAR (p < 0.001) and APRI (p = 0.007), and these scores were associated with malignancy (p < 0.005) with OR = 7.1 and OR = 5, respectively. Moreover, we showed that only in the cancer group, low levels of vitamin D correlated with stigmata of impaired metabolism.

**Discussion:**

In our study, AAR and APRI scores were associated with thyroid nodule malignancy and could be used to predict it and to speed up the diagnostic process. From a pathogenic point of view, we speculated that metabolic-associated fatty liver disease (MAFLD) along with hyperglycemia and vitamin D deficiency may represent putative drivers of thyroid carcinogenesis.

## 1 Introduction

The prevalence of palpable nodules is 5% in women and 1% in men living in iodine-sufficient parts of the world (1, 2), and an increase in thyroid cancer incidence, the most common endocrine neoplasm, has been observed in the last decades (3). In addition to familiarity and exposure to ionizing radiation, lifestyle changes and dietary habits play a significant role in the development of thyroid malignancy (4), and it has been even suggested that a healthier lifestyle may attenuate the deleterious influence of genetics on the risk of thyroid cancer (5). Indeed, obesity, diabetes, and metabolic syndrome (MetS) are characterized by a highly inflammatory microenvironment predisposing or promoting genetic instability, which underlies neoplastic transformation (6), consequently leading to increased thyroid cancer risk (7, 8). Fine-needle aspiration biopsy/cytology (FNAB/C) is the only procedure to discriminate benign from malignant nodules. Unfortunately, due to its invasiveness, it cannot be considered a screening tool. Thus, other non-invasive highly specific predictors of malignancy may be useful in clinical practice.

Similarly, liver biopsy is not feasible in all patients suspected of metabolic-associated fatty liver disease (MAFLD), the hepatic manifestation of adiposity that ranges from steatosis to liver fibrosis. Assessing the grade of liver fibrosis is critical because it can still be reversed via healthier dieting and lifestyle before progressing toward cirrhosis and hepatocellular carcinoma (9). For these reasons, nowadays, a number of non-invasive scores have gained validity as first-line tools in patients with hepatic fibrosis since they are immediate and not operator-dependent and can be used in the outpatient setting, differently from liver biopsy and transient elastography (10). Intriguingly, these scores have been proposed not only in the assessment of liver fibrosis *per se* but also as indirect markers of cardiovascular risk and diabetes (11, 12) and in predicting the development of MAFLD complications (13) and hepatic and extra-hepatic cancers (14). Aspartate aminotransferase (AST) to alanine aminotransferase (ALT) ratio (AAR), aspartate aminotransferase to platelet ratio index (APRI), and fibrosis index based on four factors (FIB-4) are among the most validated non-invasive indexes.

Hence, given the possible pathogenic association of adiposopathy with thyroid cancer onset, we considered a consecutive sample of 142 patients with newly diagnosed thyroid nodules who underwent FNAB/C with the aim of detecting any dysmetabolism-associated biomarker that might be associated with malignancy.

## 2 Materials and methods

### 2.1 Study population

Patients’ recruitment, anthropometric, biochemical, and clinical variables were registered continuously in the electronic health register of Metabolic Diseases of the Department of Interdisciplinary Medicine—Internal Medicine Division—”Aldo Moro” University of Bari (Bari, Italy) from January 2018 to September 2022. Participants underwent physical examination, biochemical assessment, and thyroid ultrasound. The study was conducted in a group of 142 consecutive outpatients (47 men and 95 women) who were diagnosed with thyroid nodules and underwent ultrasound-guided FNAB/C due to suspicion of malignancy according to ultrasound characteristics (1). Patients with acute diseases and neoplastic diseases with recent onset (less than 10 years) and/or under chemotherapy were excluded. The study was approved by the Ethics Committee (n.311, MSC/PBMC/2015) of the Azienda Ospedaliero-Universitaria Policlinico di Bari (Bari, Italy) in accordance with the requirements of the Declaration of Helsinki. Written informed consent for the use of clinical data was obtained from all participants in the study. In accordance with the approval of the Ethics Committee, only patients who were already 18 years or older were included.

### 2.2 Clinical and biochemical assessment

All participants underwent a detailed anamnesis and physical examination. Anthropometric assessment was performed using standardized procedures. To sum up, waist circumference (WC) was measured at the midpoint between the inferior part of the 12th costa and the anterior–superior iliac crest. Body mass index (BMI) was computed as weight (kg) divided by the height squared (m^2^), and BMI values (kg/m^2^) between 25 and 29.9 and over 30.0 were considered as overweight and obesity conditions, respectively. Morning blood samples were obtained from the antecubital veins after 12 hours of fasting. After blood clotting and centrifugation, serum was processed for analysis of biochemical markers of glucose and lipid metabolism. Liver and thyroid markers were also studied following standardized biochemical procedures. All biochemical measurements were centralized and performed in the ISO 9001-certified laboratories of the University Hospital of Bari. Diabetes mellitus (DM) was diagnosed according to international criteria: HbA1c (percentage of glycosylated hemoglobin) ≥6.5%, fasting plasma glucose (FPG) ≥126 mg/dl, and/or active treatment for DM. Non-invasive liver fibrosis scores were determined according to published formulas. AAR was calculated as AST divided by ALT. The cutoff value adopted was AAR < 1 for the identification of a fibrosis-free liver and AAR ≥ 1 for probable fibrosis. APRI score was calculated as AST/platelet count (×10^9^/L) × 100. The cutoff values were as follows: APRI < 0.5 for no fibrosis, APRI ≥ 0.5 for liver fibrosis, and APRI ≥ 1.5 for probable cirrhosis. FIB-4 index was calculated as age × AST/platelet count (×10^9^/L) × √ALT. The cutoff values adopted were as follows: FIB-4 < 1.45 for no or moderate fibrosis, 1.45 < FIB-4 < 3.25 for moderate fibrosis, and FIB-4 > 3.25 for extensive fibrosis or cirrhosis (15).

Thyroid ultrasound was performed with an Esaote MyLab 70 Gold ultrasound system with 10–15-MHz linear transducers. Thyroid nodules were defined according to international criteria as discrete lesions within the thyroid gland radiologically distinct from the surrounding thyroid parenchyma (1). The maximal diameter of nodules was measured in three planes: longitudinal, transverse, and anteroposterior. Fine-needle aspiration (FNA) was used after ultrasound for cytological diagnosis of nodules. The cytologic assessment was performed according to the SIAPEC-SIE 2014 guidelines. Malignancy was assumed in those diagnostic categories for which surgery was the suggested action (TIR3B, TIR4, and TIR5) (16).

### 2.3 Statistical analysis

Descriptive statistical analyses of the study sample were performed, and results were expressed as mean ± standard deviation (SD) for numerical data and as counts and percentages for categorical data. Comparisons of continuous clinical variables between two groups were performed by Student’s t-test.

The receiver operating characteristic (ROC) curves were used to determine the optimum cutoff level of AAR, APRI, BMI, and FPG in discriminating malignancy of thyroid nodules. Empirical ROC curves were plotted for these variables along with a calculation of the area under the curve (AUC) with 95% confidence intervals and one-sided upper p-values for the null hypothesis AUC = 0.5. Youden’s index, or equivalently, the highest sensitivity + specificity, was used to determine the optimal cutoff of each variable for the prediction of malignancy.

Contingency tables, chi-squared test, and Fisher’s exact test, if required, were used to study the association between categorical variables. The results were expressed as odds ratios with their relative 95% confidence interval (95% CI) and were graphically plotted in a forest plot.

Correlations among continuous variables were also analyzed and estimated using Spearman’s correlation coefficient (r). p-Values (p) lower than 0.05 were considered statistically significant. All analyses were performed using the NCSS 12 Statistical Software, version 12.0.2018 (NCSS, LLC Company, Kaysville, UT, USA) and GraphPad Prism, version 9.1.0 (GraphPad Software, San Diego, CA, USA).

## 3 Results

### 3.1 Clinical characterization of the study population

A total of 142 subjects (47 men and 95 women) with putative risk of metabolic diseases underwent thyroid ultrasound exam and were diagnosed with suspected malignant thyroid nodules, thus FNAB/C was performed. After FNAB/C, 121 patients (85%; 39 men and 82 women) were diagnosed with benign thyroid nodules, while 21 individuals (15%) were diagnosed with thyroid cancer (8 men and 13 women). As shown in **Table 1**, 106 (75%) patients had pathological WC according to the International Diabetes Federation (IDF) criterion for MetS diagnosis (17). A total of 71 patients (50%) were overweight (BMI ≥ 25 kg/m^2^), and 23 (16%) were obese (BMI ≥ 30 kg/m^2^). Moreover, 28 patients (20%) were affected by diabetes mellitus.

**Table 1:** Clinical characterization of the study population.

Study population	N = 142 (M = 47; F = 95)
WC positive criterion	106 (75%)
BMI ≥ 25 kg/m^2^	71 (50%)
BMI ≥ 30 kg/m^2^	23 (16%)
Diabetes mellitus diagnosis	28 (20%)
Benign thyroid nodules	121 (85%)
Malignant thyroid nodules	21 (15%)

WC positive criterion for metabolic syndrome diagnosis was ≥80 cm in women and ≥94 cm in men. Diabetes mellitus was diagnosed according to international criteria: glycosylated hemoglobin (HbA1c) ≥6.5%, fasting plasma glucose (FPG) ≥126 mg/dl, and/or treatment for diabetes. M, male; F, female; WC, waist circumference; BMI, body mass index.

### 3.2 Clinical and biochemical variables characterizing patients with nodule malignancy

With the aim of identifying metabolic biomarkers associated with thyroid nodule malignancy, we compared groups of patients with benign and malignant nodules. First of all, we investigated whether sex could influence thyroid cancer susceptibility. We performed a chi-squared test finding that there was no significant association (p = 0.598, **Supplementary Table 1**). Age as well as WC did not significantly differ between the two groups, while patients with thyroid cancer exhibited increased BMI (p = 0.048) and FPG levels (p = 0.046). Lipid profile, 25-OH vitamin D, and thyroid function markers were not found to be statistically different between the two groups. Regarding transaminase levels, only AST was found to be significantly higher in the malignant group (p < 0.001) compared to subjects with benign nodules, albeit in the normal range of values. Intriguingly, considering non-invasive scores for liver fibrosis, subjects with thyroid cancer presented increased AAR (p < 0.001) and APRI scores (p = 0.007), while no difference was found for FIB-4 (**Table 2**).

**Table 2:** Comparison of biochemical and clinical variables between subjects with benign nodules and malignant nodules.

	Benign nodules (n = 121)	Malignant nodules (n = 21)	p-Value
Age (years)	56.1 ± 12.8	54.1 ± 12.7	0.514
WC (cm)	92.4 ± 11.7	95.0 ± 11.6	0.345
BMI (kg/m^2^)	25.3 ± 3.8	27.2 ± 4.9	0.048*
FPG (mg/dl)	90.1 ± 13.1	97.7 ± 26.9	0.046*
HbA1c (mmol/mol)	36.3 ± 5.1	38.9 ± 9.4	0.098
Total cholesterol (mg/dl)	190.6 ± 36.8	183.7 ± 44.6	0.447
HDL-c (mg/dl)	60.1 ± 15.1	60.5 ± 17.9	0.903
LDL-c (mg/dl)	109.8 ± 31.1	103.7 ± 32.6	0.409
Triglycerides (mg/dl)	100.3 ± 50.3	100.5 ± 51.3	0.983
25-OH vitamin D (ng/ml)	26.9 ± 11.0	22.7 ± 11.1	0.143
TSH (mUI/L)	4.2 ± 24.3	2.3 ± 1.1	0.761
fT4 (ng/dl)	2.1 ± 11.8	1.0 ± 0.2	0.707
fT3 (ng/dl)	2.8 ± 0.3	2.7 ± 0.4	0.362
AST (U/L)	22.9 ± 7.4	30.7 ± 11.6	<0.001*
ALT (U/L)	28.3 ± 14.5	28.9 ± 11.8	0.863
GGT (U/L)	28.0 ± 24.6	24.9 ± 13.1	0.574
AAR	0.89 ± 0.3	1.2 ± 0.5	<0.001*
FIB-4	1.2 ± 0.7	1.4 ± 0.7	0.203
APRI	0.3 ± 0.2	0.4 ± 0.2	0.007*

Data are presented as mean ± SD (standard deviation). Comparisons were performed by Student’s t-test, and statistical significance was assessed for p-values <0.05 (*). WC, waist circumference; BMI, body mass index; FPG, fasting plasma glucose; HbA1c, glycosylated hemoglobin; HDL-c, high-density lipoprotein cholesterol; LDL-c, low-density lipoprotein cholesterol; TSH, thyroid-stimulating hormone; fT4, thyroxine; fT3, triiodothyronine; AST, aspartate aminotransferase; ALT, alanine aminotransferase; GGT, gamma-glutamyl transpeptidase; AAR, AST to ALT ratio; FIB-4, fibrosis index based on four factors; APRI, AST to platelet ratio index.

### 3.3 Discrimination of nodule malignancy by metabolic biomarkers

We performed ROC curve analyses of AAR, APRI score, BMI, and FPG to define cutoff values for discrimination of thyroid nodule malignancy.

**Figure 1:**
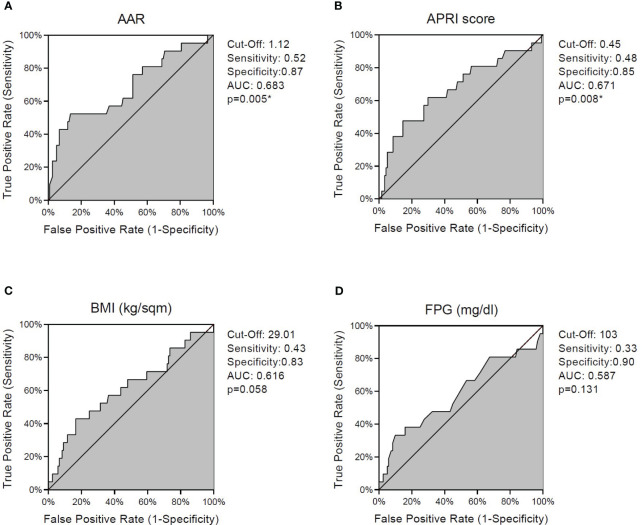
ROC curves for discrimination of thyroid nodule malignancy. Graphs indicate cutoff values with respective sensitivity and specificity levels and empirical estimation of area under curve (AUC) for AAR **(A)**, APRI score **(B)**, BMI **(C)**, and fasting plasma glucose **(D)**. (*) indicates significant p-values. ROC, receiver operating characteristic; AST, aspartate aminotransferase; ALT, alanine aminotransferase; AAR, AST to ALT ratio; APRI score, AST to platelet ratio index; BMI, body mass index; p, p-value.

The empirical ROC curve of AAR (**Figure 1A**) was characterized by the largest AUC as well as the highest Youden’s index (sensitivity = 52%, specificity = 87%), with a cutoff value of 1.12 (p < 0.01). APRI score (**Figure 1B**) presented intermediate sensitivity and high specificity (48% and 85%, respectively), with a significant AUC (p < 0.01) and a cutoff value of 0.45. The empirical ROC curve of BMI (**Figure 1C**) showed high specificity (83%) but lower sensitivity than AAR and APRI scores in discriminating thyroid cancer, with a cutoff value of 29.01 (p = 0.058). Despite very high specificity (90%), FPG levels showed the lowest sensitivity (33%) and the smallest AUC (p = 0.131) in discriminating between benign and malignant nodules, with a cutoff value of 103 mg/dl (**Figure 1D**). Overall, these data highlight the strong ability of AAR and APRI to accurately discriminate thyroid nodule malignancy.

### 3.4 Dysmetabolic condition-associated risk of malignancy

**Figure 2:**
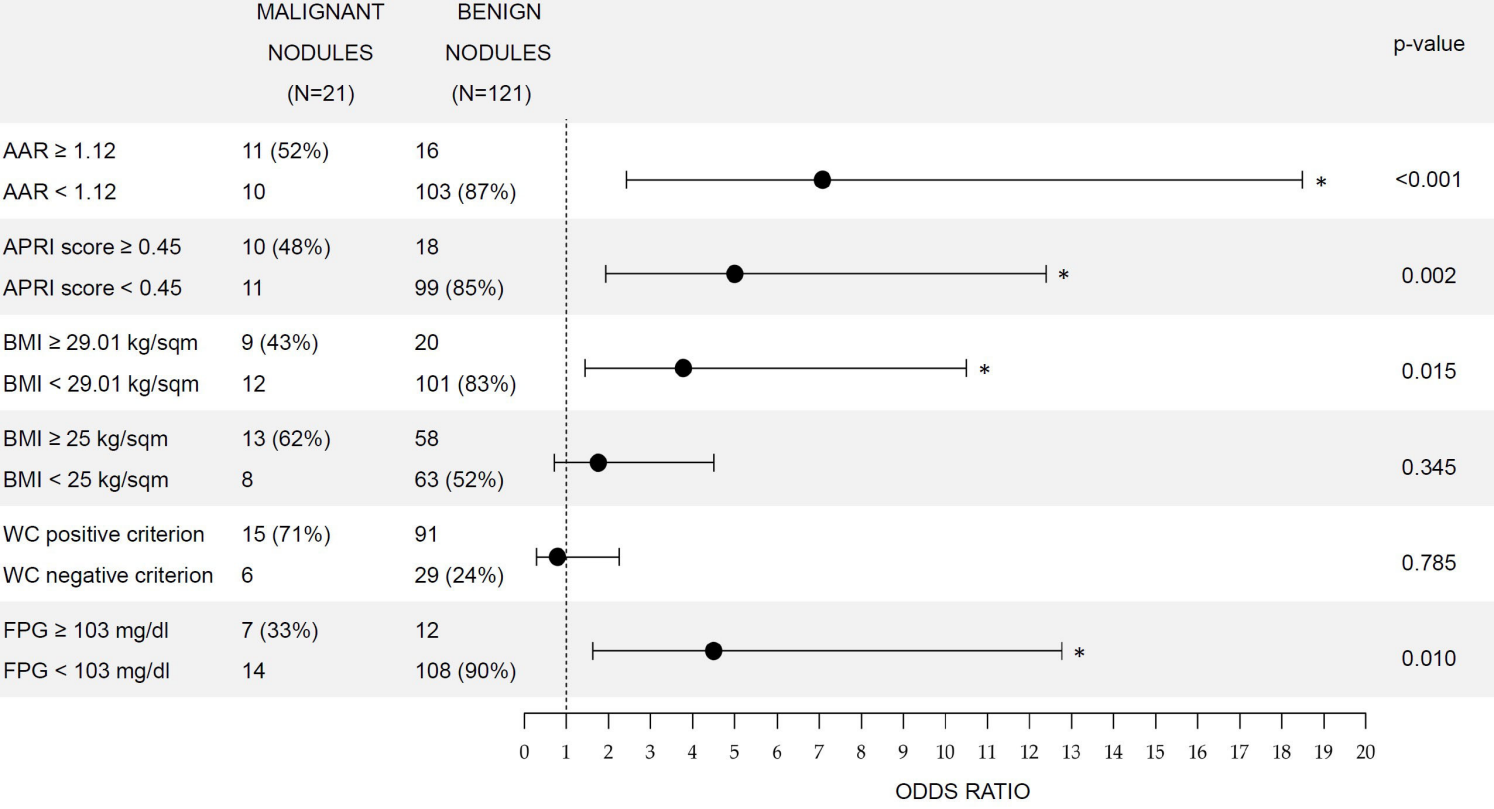
Comparison of odds ratio for thyroid cancer in patients with increased biomarkers. Contingency tables are presented on the left side of the picture. (%) in the second and third columns indicate, respectively, sensitivity and specificity. p-Value refers to chi-squared tests and (*) indicates significant p-values. Bars represent 95% CI for ORs. WC positive criterion for metabolic syndrome diagnosis was ≥80 cm in women and ≥94 cm in men. ORs, odds ratios; AST, aspartate aminotransferase; ALT, alanine aminotransferase; AAR, AST to ALT ratio; APRI score, AST to platelet ratio index; BMI, body mass index; WC, waist circumference; FPG, fasting plasma glucose.

To understand whether and to what degree dysmetabolic conditions increased the risk of thyroid nodule malignancy, we calculated the odds ratio (OR) of main biomarkers, stratifying our population according to both internationally validated cutoff values and cutoff values that have been calculated through the analysis of our ROC curves even if not significant (**Figure 2**) (18).

Values of AAR above the international cutoff for the identification of liver fibrosis (AAR ≥ 1) indicate increased risk of malignancy with an OR of 2.13 (95% CI: 1.2–3.8), although the association in the chi-squared test was not significant (p = 0.077), while AAR values above the ROC cutoff of 1.12 showed the highest OR (7.1, 95% CI: 2.4–18.5), with a significant association (p < 0.001). Considering APRI, values above the international cutoff of 0.5 and the ROC cutoff of 0.45 were both associated (p < 0.05) with malignancy, with ORs of 6.6 (95% CI: 2.0–20.0) and 5 (95% CI: 1.9–12.6), respectively.

Regarding BMI, overweight condition (BMI ≥ 25 kg/m^2^) was not associated with thyroid cancer (p = 0.345). Conversely, systemic obesity (BMI ≥ 30 kg/m^2^) was associated (p = 0.047) with a risk of nodule malignancy (OR = 3.3, 95% CI: 1.2–8.9). Interestingly, an even higher risk (OR = 3.8, 95% CI: 1.5–10.5) was associated (p = 0.015) with BMI values above the ROC cutoff of 29.01 kg/m^2^. Surprisingly, visceral obesity assessed by WC was not associated (p = 0.785) with the risk of thyroid cancer in our study population.

Finally, FPG levels above the ROC cutoff value of 103 mg/dl were also found to significantly (p = 0.01) increase the risk of malignant nodules (OR = 4.5, 95% CI: 1.6–12.8).

### 3.5 Metabolic correlations among biomarkers in benign and malignant nodule populations

**Figure 3:**
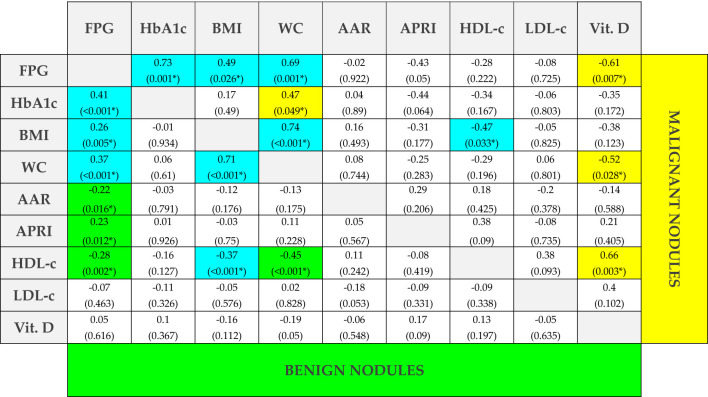
Spearman’s correlations among clinical and biochemical biomarkers in subjects with benign and malignant nodules. R and p-values (in brackets) of Spearman’s correlations among clinical and biochemical biomarkers. Statistical significance was assessed for p-values <0.05 (*). Blue cells represent correlations that are significant in both groups, yellow cells represent correlations that are significant only in patients with malignant thyroid nodules, and green cells represent correlations that are significant only in patients with benign nodules. FPG, fasting plasma glucose; HbA1c, glycosylated hemoglobin; BMI, body mass index; WC, waist circumference; AAR, AST to ALT ratio; APRI score, AST to platelet ratio index; HDL-c, high-density lipoprotein cholesterol; LDL-c, low-density lipoprotein cholesterol; Vit. D, 25-hydroxy vitamin D.

To further disclose how dysmetabolic conditions could affect the nature of thyroid nodules, we performed correlations among AAR, APRI, and biochemical metabolic biomarkers in patients with benign nodules and with cancer, separately (**Figure 3**).

**Figure 4:**
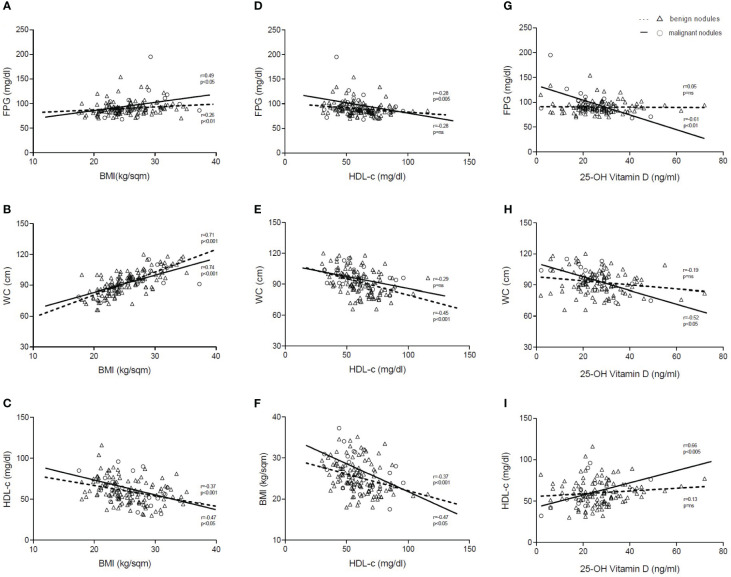
Correlations of BMI, HDL-c, and 25-OH vitamin D. Representation of Spearman’s correlations (r) of BMI **(A–C)**, HDL-c **(D–F)**, and 25-OH vitamin D **(G–I)** in patients with benign and malignant thyroid nodules. Statistical significance was assessed for p-values <0.05. Dashed lines indicate patients with benign nodules (Δ), and solid lines indicate patients with malignant nodules (o). FPG, fasting plasma glucose; WC, waist circumference; HDL-c, high-density lipoprotein cholesterol; BMI, body mass index; p, p-value; ns, not significant.

Regarding AAR and APRI, in the benign group, they both significantly (p < 0.05) correlated with FPG, although inversely (r = −0.22) and directly (r = 0.23), respectively. These results are in line with previous studies in subjects with a risk of diabetes (19, 20). Intriguingly, these correlations were both lost when considering cancer patients, suggesting that a metabolic derangement could be present in patients with cancer. To further investigate the other pathways that may be involved in cancer pathogenesis, we performed correlations among the other metabolic biomarkers. We found significant correlations of BMI with FPG (**Figure 4A**), WC (**Figure 4B**), and high-density lipoprotein cholesterol (HDL-c) (**Figure 4C**) in subjects with benign nodules, as well as in those with malignant ones. Furthermore, HDL-c showed statistically significant correlations with FPG (**Figure 4D**) and WC (**Figure 4E**) only in subjects with benign nodules, while HDL-c levels were observed to significantly correlate with BMI (**Figure 4F**) in both groups. Conversely, 25-OH vitamin D significantly correlated with FPG (**Figure 4G**), WC (**Figure 4H**), and HDL-c (**Figure 4I**) only in patients with malignant nodules, thus suggesting that its deficiency could play a crucial role in the development of thyroid malignancy.

## 4 Discussion

In this study, we show for the first time that increased non-invasive liver fibrosis scores AAR and APRI are associated with thyroid cancer in patients with newly diagnosed thyroid nodules.

Although previous studies had investigated the possible role of AAR in predicting cancer incidence and mortality, to the best of our knowledge, this is the first study specifically pointing to the possible association between thyroid cancer and liver fibrosis indexes. Indeed, in a prospective Chinese study, baseline AAR was recently proposed for the prediction of the future development of cancer in a cohort of 9,946 participants, but unfortunately, no data were supplied for the specific cohort of thyroid cancer patients (21). Also, a retrospective Japanese study in 85,658 individuals investigated whether AAR may predict cancer onset, finding that a higher AAR increased the risk of cancer development, but only in men who were drinkers, while no association was detected in women and abstainer men. However, they did not register an appropriate number of thyroid cancer cases to speculate about specific correlations (22). Furthermore, similarly to a previous study exploring APRI association with thyroid autoimmune diseases (23), we confirm the usefulness of these scores in predicting a wide range of conditions, beyond liver fibrosis. However, there are some issues to be addressed.

In order to understand the reasons for this association, the first question is whether these scores possess a predictive and diagnostic power *per se* or if their increase mirrors the pathogenic involvement of MAFLD and liver fibrosis in thyroid carcinogenesis. Indeed, a recent study enrolling 352,911 participants showed that the presence of fatty liver was a significant risk factor for the development of thyroid cancer (24). It has been hypothesized that, as soon as hepatic steatosis progresses, leptin release may cause the death of macrophages and hepatocytes through CD8+ T lymphocytes, which could in turn promote the proliferation and invasiveness of tumors such as thyroid cancer (25).

Moreover, the loss of AAR and APRI correlations with FPG in patients with cancer underscores the involvement of other dysmetabolic conditions associated with fatty liver, such as obesity, impaired glycemia, and dyslipidemia.

In line with previous studies (26–30), we observed that BMI and increased FPG were both associated with malignancy. Chronic hyperglycemia has been observed to directly influence cancer cell growth through the promotion of oxidative stress (31), and increased reactive oxygen species (ROS) concentrations have been observed in thyroid cancer cells (32). We previously identified increased visceral adipose tissue and oxidative stress as predisposing conditions to impaired reverse cholesterol transport (RCT), a process in which HDL particles are critically involved (33). In this study, HDL level intriguingly shows significant correlations with FPG and WC only in subjects with benign nodules, while a significant direct correlation with 25-OH vitamin D, as previously reported (34, 35), was found only in patients with malignant nodules. On the one hand, the paradoxical uncoupling of cholesterol bioavailability and synthesis has already been shown in cancer cells (36). On the other hand, although the association of vitamin D levels with thyroid cancer onset is still being debated, our result strengthens the hypothesis that elevated serum levels of vitamin D hold a protective role toward thyroid malignancy (37) by inhibiting pro-tumorigenic inflammation (38). Nevertheless, given its role in the regulation of intestinal barrier integrity and control of innate and adaptive immunity in the gut (39), our result also suggests that lower levels of vitamin D may contribute to a worsening of the alterations of the gut microbiota typically observed in dysmetabolic subjects, thus tightening the relationship between metabolic diseases and thyroid cancer. Thus, one should consider that low vitamin D levels could increase the extra-hepatocytic inflammation process that dumps the progression of steatosis to fibrosis, also opening new perspectives on the utility of vitamin D supplementation in the prevention and treatment of MAFLD and supporting the metabolic interplay among hepatocyte infarction, imbalanced cholesterol metabolism, and cancer (40).

Our study has some limitations. First, the small size of our sample underlines the pressing need for further validation of AAR and APRI in a different cohort of metabolic subjects undergoing FNAB/C. Second, our data lacked potential important confounders, such as the use of antidiabetic and lipid-lowering drugs, which may influence the development of steatohepatitis and liver function, as well as anti-platelet treatment that may influence platelet count used for the calculation of APRI. Third, data on environmental risk factors and cancer susceptibility (41) were self-reported by patients, and no further detailed information was available. Finally, non-invasive scores should be correlated with liver ultrasound and elastography data to assess whether they represent the stigmata of liver dysfunction that eventually acts as a carcinogenesis trigger.

In conclusion, the strength of our study lies in the ability of non-invasive liver fibrosis scores AAR and APRI to assess the risk of malignancy in thyroid nodules of metabolic subjects. Thereby, on the one hand, in the future, exploring this association might lead to elaborate new scores to predict thyroid malignancy and to speed up the diagnostic process, also targeting patients who need to be offered FNAB/C of suspected nodules. On the other hand, future studies are needed to prove that reducing obesity, counteracting fatty liver-associated diseases and metabolic derangements, and supplementing vitamin D could probably prevent thyroid cancer onset at least in specific groups of patients.

## Data availability statement

The raw data supporting the conclusions of this article will be made available by the authors, without undue reservation.

## Ethics statement

The studies involving humans were approved by the Ethics Committee (n.311, MSC/PBMC/2015) of the Azienda Ospedaliero-Universitaria Policlinico di Bari (Bari, Italy). The studies were conducted in accordance with local legislation and institutional requirements. The participants provided their written informed consent to participate in this study.

## Author contributions

Conceptualization, AM; methodology, LC, GG, and AM; software, LC, FN, and CM; formal analysis, FN, CM, SP, and GG; investigation, PS, EB, GA, GP, and CS; resources, LC and FN; data curation, FN, CM, and SP; writing—original draft preparation, FN; writing—review and editing, LC and AM; visualization, LC and FN; supervision, AM; project administration, AM; funding acquisition, LC, PS, and AM. All authors have read and agreed to the published version of the manuscript.
