# Supplementary material for: Thyroid nodule malignancy is associated with increased non-invasive hepatic fibrosis scores in metabolic subjects
Source: Front Oncol. 2023 Oct 26;13:1233083. doi: 10.3389/fonc.2023.1233083 (PMC10641401; doi:10.3389/fonc.2023.1233083)
Supplement: Supplementary file 1 [file DataSheet_1.pdf]

Supplementary table 1. Association of sex and malignancy in thyroid nodules.

	**Benign nodules (N=121)**	**Malignant nodules (N=21)**
**Females (N=95)**	82 (67.8%)	13 (61.9%)
**Males (N=47)**	39 (32.2%)	8 (38.1%)

The association was evaluated with Chi-squared test and was found not significant (p-value=0.598) Sensitivity was 67.8%; specificity was 38.1%. (%) indicate the percentage of column total.
